# Membrane-destabilizing ionizable phospholipids: Novel components for organ-selective mRNA delivery and CRISPR–Cas gene editing

**DOI:** 10.1038/s41392-021-00642-z

**Published:** 2021-05-25

**Authors:** Ning Meng, Dirk Grimm

**Affiliations:** 1 Department of Infectious Diseases/Virology, Medical Faculty, University of Heidelberg, Heidelberg, Germany; 2 BioQuant, University of Heidelberg, Heidelberg, Germany; 3 German Center for Infection Research (DZIF) and German Center for Cardiovascular Research (DZHK), partner site Heidelberg, Heidelberg, Germany

**Keywords:** Gene delivery, Drug delivery, Gene delivery

In a new study published in *Nature Materials*, Liu et al.^1^ report a novel design of lipid nanoparticles (LNPs) in which multi-tailed ionizable phospholipids (iPhos) constitute the active component, and which facilitates endosomal escape and thus improves delivery of mRNA and/or single-guide (sg)RNA for in vivo gene editing. LNPs composed of the best-performing iPhos and different helper lipids—zwitterionic lipids, ionizable cationic lipids and permanently cationic lipids—achieved selective organ targeting (SORT) and organ-specific CRISPR-Cas9 gene editing in spleen, liver, and lungs of mice, respectively.^1^

**Fig. 1:**
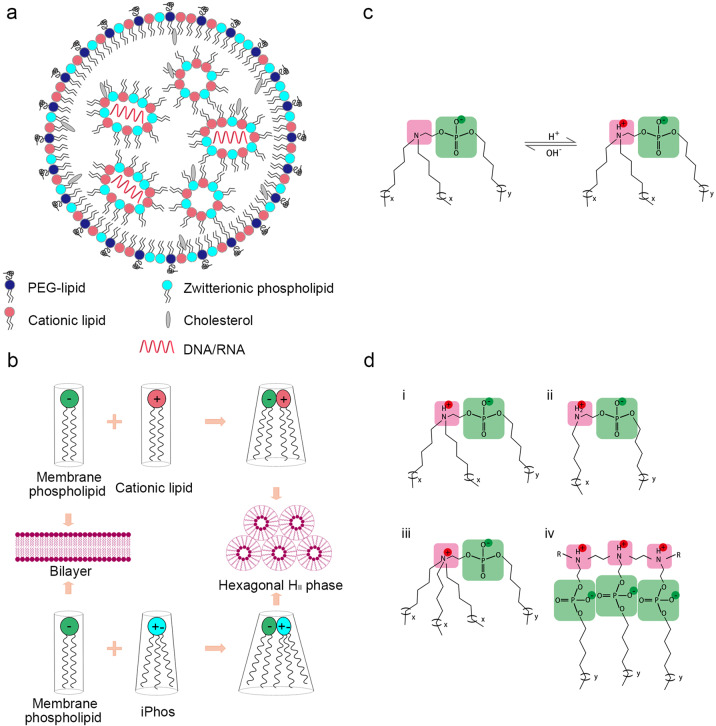
Structure and function of designed iPhos containing ionizable amines. **a** Structure and composition of LNP. **b** Proposed mechanism of cationic lipid and iPhos-mediated membrane disruption and endosomal escape. Adapted from Liu et al.^1^ and Semple et al.^4^ **c** Rationally designed iPhos with one ionizable amine, one phosphate group, and three alkyl tails (x and y indicate the carbon lengths). The ionizable amine is neutral at physiological pH, but positively charged in the endosome. Adapted from Liu et al.^1^ **d** Four classes of iPhos. See text for details

A main challenge in DNA/RNA-based gene therapy is the delivery of nucleic acid molecules into target cells. To enter cells, these molecules need to be encapsulated into specialized vectors as the cell membrane is inherently not penetrable by naked DNA or RNA. Over the last decade, non-viral vectors have attracted increasing attention owing to their ability to deliver and/or co-deliver different cargos for gene therapy (DNA, siRNA, mRNA, etc), as well as their ease of manufacturing, mild immunogenicity, relatively low toxicity, and compatibility with repeated dosing.^2,3^ Among the available non-viral vector variants, LNPs may be the most developed. They are typically composed of multiple entities, including (1) cationic lipids containing ionizable amines. The latter is positively charged at low pH during LNP manufacturing to facilitate encapsulation of negatively charged DNA/RNA. In contrast, they are relatively neutral at physiological pH to avoid the formation of large complexes (previously observed in LNPs using permanent cationic lipids), and protonated again in the endosome to induce endosomal escape. Moreover, LNPs comprise (2) zwitterionic phospholipids as helper or structure lipids that mimic lipids in cell membranes, (3) polyethylene glycol (PEG) lipid to provide a hydrating layer surrounding the nanoparticle, and (4) cholesterol to stabilize the nanoparticle (Fig. 1a).^2^

Despite recent progress in LNP design, the efficiency of RNA/DNA delivery by the current LNP generation remains rather low. LNPs enter cells by endocytosis and are then routed into the endosome. From there, only 1–4% of LNPs can escape and release DNA/RNA into the cytosol, largely explaining the low LNP efficiency. A proposed mechanism of endosomal escape of LNPs is that interaction between cationic lipids in the LNP with anionic lipids of the endosome membrane induces non-bilayer hexagonal H_II_ phase formation, leading to disruption of the endosome membrane (Fig. 1b).^3,4^ Previous studies have mainly focused on optimizing ionizable cationic lipids, while zwitterionic phospholipids were merely regarded as helper lipids needed to form LNPs and remained unexplored. Intriguingly, though, zwitterionic phospholipids resemble the lipids forming the endosome membrane, implying their potential to fuse into the endosome membrane and thus trigger membrane disruption.

Guided by this hypothesis and by their experience from cationic lipid optimization, Liu et al. rationally designed a series of multi-tailed, ionizable zwitterionic phospholipids called iPhos, which contain an ionizable amine, a phosphate group and three hydrophobic alkyl tails (Fig. 1c). Like ionizable cationic lipids, ionizable amines in iPhos mediate pH-dependent membrane disruption. At physiological pH, the amine group is not protonated and the iPhos with the negatively charged phosphate groups cannot fuse into the anionic biological membrane. In contrast, at the low pH in the endosome, the amine group is positively charged to form a zwitterionic head together with a phosphate group, which is able to interact with the endosome membrane and to form the H_II_ phase (Fig. 1b, c).^1^

In an initial screen, the authors evaluated mRNA delivery in vitro and therefore designed four classes of iPhos, which were mixed with simple ionizable cationic helper lipid *N*-methyldioctadecylamine (MODA), cholesterol, and 1,2-dimyristoyl-rac-glycero-3-methoxy(poly(ethylene glycol-2000)) (DMG-PEG2000) (25:30:30:1 mol/mol) to formulate iPhos LNPs (iPLNPs). Notably, while iPLNPs contain the same four components as typical LNPs, the key component that was optimized in this work is iPhos instead of cationic lipid. For functional validation, the iPLNPs were then harnessed to deliver luciferase mRNA into the ovarian cancer cell line IGROV1. As expected, iPhos with single ionizable zwitterionic head and three tails (Fig. 1d, i) showed the highest efficiency, while iPhos with single ionizable zwitterionic head and two tails (Fig. 1d, ii) were less potent as their small tail prevents the formation of the cone shape needed to induce the H_II_ phase. Moreover, iPhos with permanent zwitterionic head (Fig. 1d, iii) lacked structural flexibility on endosomal internalization, and iPhos with multiple zwitterions (Fig. 1d, iv) inefficiently inserted into the endosome membrane due to their large head.^1^

To verify that the well-performing iPhos induce endosomal escape by the membrane disruption mechanism, the authors investigated phase transformation by using ^31^P NMR spectroscopy. Indeed, a peak was observed representing the hexagonal H_II_ phase after mixing endosomal mimicking liposome and iPLNPs. iPhos-induced membrane disruption was additionally supported by data from a hemolysis model and by a fluorescence resonance energy transfer (FRET) assay.^1^

A general concern in vector research is translation of in vitro vector functionality to an in vivo setting. To address this, Liu et al. selected 51 iPhos variants from an in vitro screening for an in vivo study. This important experiment confirmed that iPhos with single ionizable zwitterionic head and three tails are also the most efficacious in vivo at both, low (0.1 mg/kg) or high mRNA dose (0.25 mg/kg). The authors moreover compared the best performing iPhos 9A1P9 with commonly used phospholipids 1,2-dioleoyl-sn-glycero-3-phosphoethanolamine (DOPE) and 1,2-distearoyl-sn-glycero-3-phosphocholine (DSPC), and showed 40- to 965-fold higher in vivo efficiency, which confirmed that the enhanced iPLNP transfection efficiency was caused by iPhos but not by any other components. Interestingly, although the mechanism was unclear, alkyl chain length mediated efficacy and selectivity. In detail, 8–10 carbon lengths at the amine side showed highest efficacy, while carbon lengths at the phosphate group affected selectivity, with 9-12 showing liver tropism and 13-16 carbon length showing spleen tropism.^1^

To date, most LNPs only showed efficient delivery to parenchymal liver cells, *i.e*., hepatocytes. Interestingly, in prior work, the authors had developed SORT LNPs and hypothesized that internal charge may be key for tissue tropism. Indeed, they found that zwitterionic/anionic, ionizable cationic, or permanently cationic lipids can lead to selective mRNA delivery to spleen, liver, or lungs, respectively.^5^ Hence, Liu et al. now combined iPhos and their SORT method, by mixing their best-performing iPhos 9A1P9 with zwitterionic lipids (DOPE), ionizable cationic lipids (MDOA, 1,2-dioleoyl-3-dimethylammonium-propane (DODAP) and 5A2-SC8), or permanently cationic lipids (dimethyldioctadecylammonium bromide salt (DDAB) and 1,2-dioleoyl-3-trimethylammonium-propane (DOTAP)) as helper lipids. Notably, this enabled selective mRNA delivery to spleen, liver, and lungs in intravenously injected mice. Further encouraging with respect to clinical application is that intravenous co-delivery of Cas-mRNA/sgRNA in mice by liver-selective 9A1P9-5AS-SC8 iPLNP or lung-selective 9A1P9-DDAB iPLNP resulted in organ-specific CRISPR/Cas-mediated gene editing (total RNA dose: 0.75 mg/kg). To further confirm the great potential for clinical application, the authors showed that iPLNPs produced in large scale using microfluidic mixing retained efficient and organ-selective delivery, allowed repeat injection, and were safe at the tested doses.^1^

This study advances phospholipids from an unexplored helper component of LNPs to a key player in non-viral vector design, and thus significantly expands the repertoire of strategies for LNP optimization. The reported findings that this novel LNP design mediates highly efficient and organ-specific mRNA delivery as well as CRISPR/Cas-mediated gene editing in vivo are highly informative and promising for numerous applications including human gene therapy.
